# A compact theory of magnetic nerve stimulation: predicting how to aim

**DOI:** 10.1186/1475-925X-13-53

**Published:** 2014-04-30

**Authors:** Charles F Babbs

**Affiliations:** 1 Weldon School of Biomedical Engineering, Purdue University, 206 South Martin Jische Drive, West Lafayette, Indiana 47907-2032, USA

## Abstract

**Background:**

A compact theory that predicts quantitatively when and where magnetic neurostimulation will occur is needed as a guide to therapy, ideally providing a single equation that defines the target volume of tissue excited by single or dual coils.

**Methods:**

A first-principles analysis of magnetic stimulation incorporating a simplified description of electromagnetic fields and a simplified cable theory of the axon yields a mathematical synthesis predicting how to aim.

**Results:**

Nerve stimulation produced by a single circular coil having one or more closely packed turns occurs in donut shaped volume of tissue beneath the coil. Axons spanning several millimeters are the sites of magnetic stimulation. The sites of maximal transmembrane depolarization in nerve fibers correspond to points where the axons enter or exit this volume of magnetically induced voltage and current. The axonal membrane at one end is depolarized locally during the rising phase of current in the coil. The axonal membrane at the opposite end is depolarized locally during the falling phase of current in the coil. Penetration depths of several centimeters from the skin surface or approximately one to two coil radii are practical. With two coils placed in a figure-of-eight configuration the separate clockwise and counterclockwise currents generate magnetic fields that add, producing maximal stimulation of a spindle shaped volume, centered at a depth of one-third to one-half coil radius from the body surface.

**Conclusions:**

This condensed synthesis of electromagnetic theory and cable theories of axon physiology provides a partial solution to the targeting problem in peripheral and in transcranial magnetic stimulation.

## Background

Magnetic neurostimulation is a remarkable phenomenon. It is electrodeless, nearly painless, and dry—easily penetrating skin and bone, which usually provide high resistance barriers to current injected through ordinary contact electrodes [1-4]. Stimulating magnetic fields are produced by high intensity electric current, flowing in thick wire coils a few centimeters in diameter that are placed on or near the skin surface. Direct contact with the body surface is not needed. An air gap is perfectly acceptable. Coils placed near the skull can stimulate neurons in the cerebral cortex, a process known as transcranial magnetic stimulation or TMS [5-9]. When coils are placed over the motor strips of cerebral cortex, just above the ears, TMS can be used to test the function of motor tracts extending from head to toe or from head to fingertips [8,10,11]. Such testing can be useful in monitoring the functional integrity of the spinal cord during neurosurgery, helping to avoid the dreaded complication of postoperative paralysis [3,12]. When coils are placed further forward over pre-frontal cortex TMS can be used treat psychic depression [8].

The current-carrying coils used to produce magnetic stimulation have one major drawback: they are hard to aim, producing diffuse stimulation. To reach its full potential magnetic neural stimulation needs to be better targeted. Although mapping of the magnetic fields in three dimensions is well understood [13,14]; less work has been done to characterize the induced electric fields and eddy currents [2,4,15-17] and even less to understand quantitatively the physiological mechanisms by which nerve stimulation happens in this unusual setting [18,19]. A successful working theory should allow one to predict which neurons will be stimulated at which orientations and at which distances from the axes of the coils [19]. This latter problem requires particular attention to the effects on subthreshold membrane potentials of neurons [20]. Practitioners need to be able to better visualize which nerve fibers in which volumes of space are likely to be excited by a defined magnetic pulse from single or double coils with a defined geometry. What is needed as a guide to therapy is a compact model that predicts quantitatively when and where neurostimulation will occur—ultimately, perhaps, providing a single equation that defines a target volume of tissue.

Magnetic fields are easily mapped and computed [21], and they can be measured routinely in a dry laboratory using small search coils [22], although even the search coil method has been criticized [23]. However, the induced electric fields in volume conductors are not as easily computed or measured. Induced electrical potential gradients depend on the size and shape of any particular current loop or path and the presence of boundaries and barriers to current flow [16]. The induced electric field can be predicted from the negative of the time-derivative of the vector potential field using Finite Element Method packages such as Comsol®, but the computations are not easy for most clinical practitioners. Induced voltage gradients are also challenging to measure in the laboratory owing to electromagnetic interference from the high coil currents needed to produce the magnetic fields [23,24].

In this context a mathematical modeling approach can be insightful. Many thinkers have been attracted to the complex and interesting problems of predicting the magnetic fields created by TMS coils of various shapes as well as predicting the induced electric fields in various anatomical models. Several quite elaborate models have been described [2,15,25] to predict magnetically induced eddy currents. These are based upon complex analytical treatments or upon finite element and finite difference models implemented by computer programs, some of which taking over an hour of computer time to execute [4] and most of which taking several tens of hours of human thinking time to fully comprehend. Once understood, such rigorous and detailed solutions for specific cases are not especially transportable or portable from one patient to the next or one biological application to the next.

For many relatively simple and clinically relevant geometries a compact analytical model could allow a more intuitive understanding of the influence of dominant parameters, based on the algebraic form of the final equation. Once derived, such an equation might well encapsulate much knowledge in compact notation and still be applicable to a large number of specific cases. This approach to understanding magnetic stimulation might be especially useful to clinicians not supported by a department of biomedical engineering that is capable of numerical modeling of many individual patients.

Thus the field of magnetic neural stimulation could benefit from a relatively straightforward, working theory that provides a way to visualize the three dimensional distribution of neurons that will be stimulated by a specified external coil—in other words, a pattern that tells the user how to aim the coil to stimulate a particular set of targets, including peripheral nerves, brain cortical regions, or even deeper brain structures, including if possible the directions of the nerve fibers in space that are likely to be activated. Such a simple, working scheme for visualization is not out of reach. The general features of magnetically induced voltage gradients, sufficient for modeling the physiology of nerve stimulation, can be calculated analytically because of the circular symmetry of the problem in a relatively uniform volume conductor like brain tissue. Simple boundary conditions, such as a flat or gently curved insulating surface and unlimited depth and width can be utilized [20]. Such conditions apply to brain covered by layers of skin, skull, and cerebrospinal fluid, or peripheral nerves covered by flat skin and subcutaneous tissue. Here the mathematics of electromagnetism and the mathematics of nerve impulse generation can be combined to create an analytical model of magnetic stimulation of brain and nerves that is sufficiently accurate for medical and biological applications, yet is mathematically compact, three dimensional, and easy to visualize.

This paper presents a fresh, clean-sheet analysis along these lines to clarify the underlying physics and to answer the following questions. How do magnetic fields stimulate nerve cells? What parts of the neuron are depolarized sufficiently by magnetic stimulation to generate an action potential? Where are the zones of stimulation located in the field beneath the exciting coils? How deep can stimulation be achieved for a given coil geometry? How does the spatial orientation of the nerve fiber tracts influence the ability of nerve fibers to be stimulated? How might the use of figure of eight coil configurations better target particular brain regions for focal stimulation?

The approach followed here is to begin with first principles (1) to describe the physics by which electric currents and voltages are produced in tissue by magnetic fields and then (2) to explore the mechanisms by which action potentials are subsequently initiated in neurons. The goal is to predict the approximate three dimensional patters of magnetically induced voltage and current and also to predict in terms of easily measured variables the target volume of space in which neurons are brought from their resting membrane potential to the threshold membrane potential for initiation of action potentials.

## Theory and results

### Part one: quantitative estimates of magnetically induced electric fields

#### Electromagnetism and electromagnetic induction

**Figure 1:**
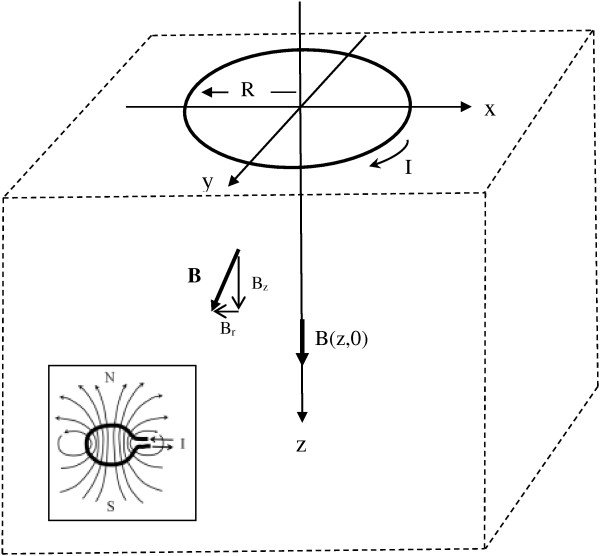
Current carrying coil and volume conductor.

High intensity electric current in simple circular coil or paired figure of eight coils on or above the skin surface produces rapidly changing magnetic fields that penetrate skin, bone, and underlying soft tissues. In the coordinate system shown in Figure 1, a current carrying wire coil of radius, R, is placed at level z = 0 and centered at the origin. The radial distance from the z-axis is $r=\sqrt{x^{2}+y^{2}}$.

In Figure 1 the vector, **B**, is the magnetic flux density in units of Tesla. For a circular coil, the magnetic or B-field induced by the coil has radial symmetry. In general, off-axis magnetic field vectors, **B**, have both axial and radial components, B_z_ and B_r_. The axial component, B_z_, is most important for magnetic neural stimulation. Along the central z-axis at x = 0, y = 0 the outward radial components, B_r_, created by current, I, flowing in paired short segments along the circumference on opposite sides of the coil cancel exactly, and so B_r_ = 0. However, the z-axis components, B_z_, created by current, I, flowing in various short segments of the circumference of the coil add or reinforce one another to induce a well characterized [14] z-axis magnetic field.

(1a) $$B\left(z,0\right)=\frac{\mu_{0}}{2}\frac{R^{2}I}{{\left(z^{2}+R^{2}\right)}^{3/2}}.$$

The constant μ_0_ is the magnetic permeability of free space in units of Henries/meter. This and other variables and mathematical symbols are defined for easy reference in Table 1, “Nomenclature”. If the coil has N closely packed turns, the result is multiplied by N. It is helpful to express tissue penetration distance, z, relative to the radius of the coil. This normalized distance is $\hat{z}=z/R$. Then the z-axis magnetic field is given by

(1b) $$B\left(\hat{z},0\right)=\frac{\mu_{0}}{2R}\frac{I}{{\left({\hat{z}}^{2}+1\right)}^{3/2}}.$$

**Table 1:** Nomenclature

**Variable**	**Units**	**Description**
a, b		Regression constants describing radii at which axial field strength falls to zero
B	Tesla	Magnetic field strength
B_z max_	Tesla	Maximal z-axis component of magnetic field
C_m_	Farads/cm^2^	Specific membrane capacitance of nerve cells
$\vec{E}$	V/m	Vector of magnetically induced electric field
E	V/m	Signed scalar magnitude of induced electric field around a circular path in homogeneous models
*E*_*L*_	V/m	Scalar component of the voltage gradient along the path of an axon
*ΔE*_*m*_	Volts	Change in transmembrane potential of an axon
I	Amps	Coil current
I_max_	Amps	Maximum coil current in time
L	m	Length of an axon segment
μ_0_	Henries/m	Magnetic permeability of free space
n		Number of coil turns or fold-increase in coil current
Φ	Tesla⋅m^2^	Magnetic flux
R	m	Coil radius
r	m	Radial distance from z-axis in space
$\hat{r}$		Normalized radial distance, r/R
R_a_	Ohms	Resistance of axoplasm
ρ	Ohm-m	Resistivity of intracellular fluid
S	m^2^	Surface area for magnetic flux
s	m	Span or width of cell
t	sec	Time
θ	Radians	Angle between induced electric field and an axon segment
|*ΔV*_*L*_|	Volts	Absolute value of voltage appearing along the length, L, of axon
x, y, z	m	Spatial coordinates
$\hat{x},\;\hat{y},\;\hat{z}$		Spatial coordinates normalized by coil radius, R
x_0,_ r_0_	m	Radial distance from z-axis at which axial component of magnetic field becomes zero

**Figure 2:**
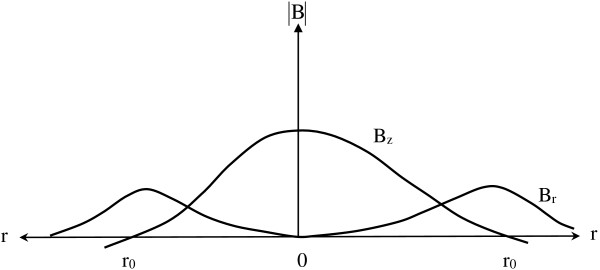
**Absolute magnitudes of axial and radial components of the magnetic field a function radial distance from the central z-axis of the coil.** Sketched from Alfonsetti et al. 2010 [21], and Cohen et al. 1990 [22].

The off-axis magnitude of the axial component, B_z_(z, r), diminishes with radial distance, r, away from the z-axis in a way characterized by much more complex equations [13], but easily appreciated graphically. In general, except when z < < R, the magnitude of B_z_(z, r) as a function of r is roughly Gaussian or dome shaped, with maximal values near r = 0, diminishing to near zero values at larger radii (Figure 2) [21,22]. Very near the plane of the coil itself (z < < R) the field is more flat [21,22] and less dome shaped. This near field region is typically outside the body during magnetic neural stimulation and its extra complexities can be ignored for present purposes.

At any particular depth, z, the magnetic field has axial and radial components, B_z_(z, r) and B_r_(z, r), sketched approximately in Figure 2 to show relative shapes and strengths. Of note are the radii r_0_, at which the axial field strength falls to zero. The values of r_0_ increase gradually as a function of depth, z. As distance, z, from the plane of the coil increases, the z-directed components of the magnetic field spread out and diminish in intensity.

#### Induced electric fields in homogeneous underlying tissues

The induction of electrical potentials in the tissue underlying the current carrying coils depends on the magnetic flux appearing through a closed loop of conducting material such as a wire, or in this case ion-containing body fluids. The magnetic flux, dΦ, through a small window of area dS is defined as the product of the area dS and the component of the magnetic field, **B**, normal or perpendicular to the plane of dS, that is

(2a) $$\mathrm{d\Phi}=B_{n}\;\mathrm{dS},$$

and the total magnetic flux

(2b) $$\Phi =\int_{S}B_{n}\;\mathrm{dS}.$$

Rapidly changing magnetic fields induce corresponding electric fields (fields of induced electromotive force, EMF, or voltage) as described by Faraday’s law, which is expressed in terms of the magnetic flux Φ, through a closed loop of conducting material. The induced electrical potential, **E**, around the loop is

(3a) $$E=-\frac{\mathrm{d\Phi}}{\mathrm{dt}},$$

the sign and direction of **E** around the loop being defined by the right hand rule. A more modern and more general statement of Faraday’s law is the integral form. If we define the electric field, $\vec{E}$ (volts/meter), along a wire or conducting loop for any shape of closed path consisting of multiple steps, $d\vec{L}$, in a time-varying magnetic field, then the result of Faraday’s law is

(3b) $$\oint \vec{E}\cdot d\vec{L}=-\frac{\mathrm{d\Phi}}{\mathrm{dt}}=-\frac{d}{\mathrm{dt}}\int_{\text{surface}}\vec{B}\cdot d\vec{S}=E,$$

where the integral is taken over the area bordered by the closed path [14]. In Figure 1 when the current, I, in the coil changes rapidly, the induced magnetic field components B_z_ are normal to the x-y plane and correspond to B_n_ in Equation (2b).

Measuring EMF induced by a rapidly changing magnetic field using a search coil in a dry laboratory is a straightforward task. One simply fashions a small loop of insulated wire about 1 cm in diameter and measures the voltage appearing between the ends of the wire. By tilting the plane of the coil one can find the direction of the field. In a volume conductor, however, the induced currents are constrained by insulated boundaries, and some further insight is required.

#### Radial components of the B-field can be ignored

**Figure 3:**
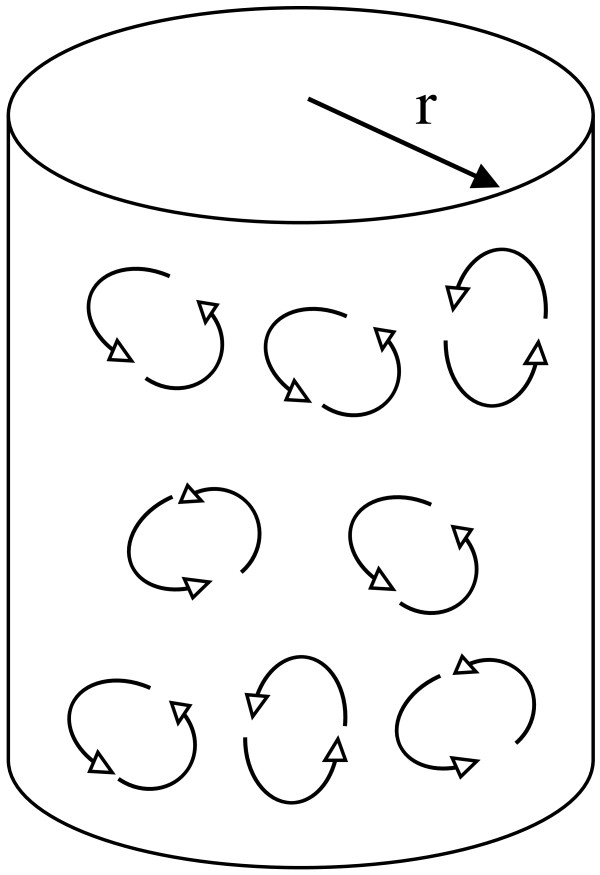
**Visualizing EMFs and currents induced by radial components of the B-field beneath a current carrying coil.** Induced currents in any potential loop tend to be cancelled by those in neighboring loops.

Figure 3 illustrates radial components of magnetic field and putative loops of induced EMF and current in a volume conductor beneath a stimulating coil. In three dimensions there are many radial directions, along which effects interact. Induced voltage and current fields in this case would tend to cancel (destructive interference). On a bounded flat plane surface the inner current loops would cancel but boundary currents would remain. However, in the continuous hoop dimension of any cylinder at radius, r, there is no boundary. Hence any voltage gradients induced by the changes in the radial components B_r_ of the magnetic field are greatly reduced by destructive interference. As a practical matter, if the plane of the coil is parallel or tangent to the body surface, the radial components B_r_(z, r) can be ignored in neural stimulation [26].

#### Estimating z-axis components of the B field

In the case of the z-axis components, B_z_(z, r), because of the radial symmetry of the problem, the differential surfaces dS in Equation (2b) are nested rings of diameters, 2r, circumference 2πr, and width, dr. The magnetic flux at any depth, z, is therefore,

(4) $$\Phi \left(z,r\right)=2\pi \int_{0}^{r_{0}}B_{z}\left(z,r\right)\;r\;\mathrm{dr}.$$

**Figure 4:**
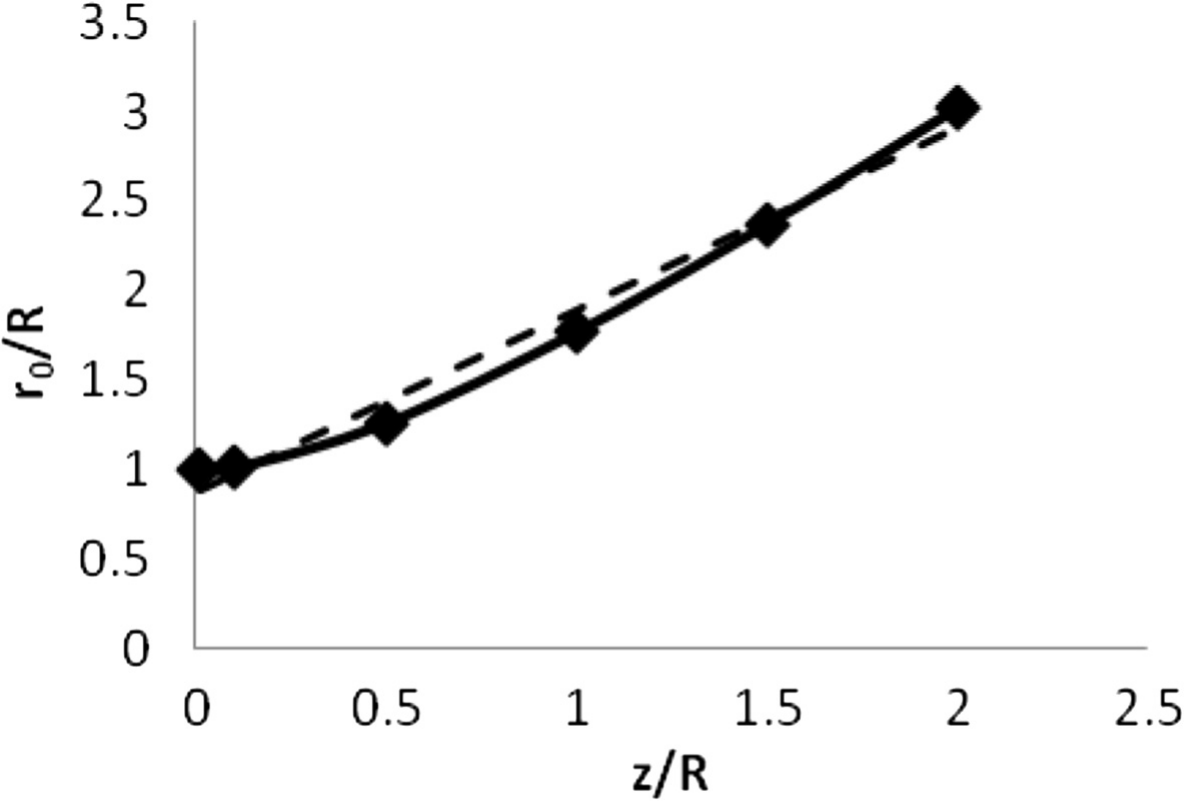
**Extent, r**_**0**_**, of the dome shaped region of positive z-directed magnetic field as a function of axial depth beneath a simple circular coil.** The linear regression function (dashed line) is y = 1.02x + 0.87.

The exact function, B_z_(z, r), describing axial field B_z_ as a function of radius r is complex and contains elliptic integrals that cannot be simply computed [13,14]. However except for cases of z < < R, the shape of B_z_(r) in three dimensions is generally a dome or bell shaped surface. In this region of biological interest, the strength of B_z_(r) falls from a maximal value at r = 0 to zero at r = r_0_[22]. The value of r_0_ gradually increases with depth as the strength of B_z_(r) weakens (Figure 4). Here the extent of the dome shaped region of positive z-directed magnetic field is computed from the exact formula [27] and plotted as a function of axial depth beneath a simple circular coil. The dashed line is the linear regression ${\hat{r}}_{0}\left(z\right)=1.0\;\left(\hat{z}+0.87\right),$ where the distances, r and z, embellished with the “hat” symbols, are normalized by the coil radius, R, so that $\hat{r}=r/R$ and $\hat{z}=z/R.$

Approximating the dome shaped function B_z_(z, r) as the parabola $B_{z}\left(z,0\right)\left(1-\frac{r^{2}}{r_{0}^{2}\left(z\right)}\right)$, in keeping with more detailed and exact computations [13,22], one can take advantage of the circular symmetry of the problem to find the total magnetic flux through circles of any radius, 0 ≤ r ≤ r_0_, centered along the z-axis and perpendicular to it. This approximation greatly simplifies the calculation of magnetic and induced electric fields and is sufficiently accurate for the purpose of understanding the biological response to magnetic stimulation. If desired, the parabolic representation of B_z_(z, r) can be expanded to a higher order polynomial in r for any desired degree of accuracy. The following approach to integration will still be valid.

For each ring of radius r and thickness dr the incremental magnetic flux is the product of field strength at radius, r, and incremental area, 2πrdr, or

(5a) $$\mathrm{d\Phi}\left(z,r\right)=B_{z}\left(z,0\right)\left(1-\frac{r^{2}}{r_{0}^{2}\left(z\right)}\right)\cdot 2\mathrm{\pi rdr}.$$

The total z-directed magnetic flux for a circular domain from r = 0 to radius r ≤ r_0_ is therefore

(5b) $$\begin{array}{ll} \Phi \left(z,r\right) & =2\pi \int_{0}^{r}B_{z}\left(z,0\right)\left(r-\frac{r^{3}}{r_{0}^{2}\left(z\right)}\right)\;\mathrm{dr}=2\pi B_{z}\left(z,0\right)\left(\frac{r^{2}}{2}-\frac{r^{4}}{4r_{0}^{2}\left(z\right)}\right)\\ & =B_{z}\left(z,0\right)\pi r^{2}\left(1-\frac{r^{2}}{2r_{0}^{2}\left(z\right)}\right), \end{array}$$

where B_z_(z,0) is given by Equation (1a). For radii r > r_0_ one can estimate the magnetic flux through a loop of electrical conductor with radius r as *Φ*(*z*, *r*) = *Φ*(*z*, *r*_0_), ignoring low amplitude reversed flux at outer radii, which are outside the region of interest in magnetic stimulation. (Alternatively, a higher order polynomial can be used.) In this way the magnetic flux through a circular domain of the volume conductor with radius r can be estimated from the peak on-axis value in Equation (1), multiplied by the area of the circle and the attenuation factor in parentheses in Equation (5b).

Further, one can estimate the rate of change in magnetic flux during the rising phase of current flow in the coil as the maximal value divided by the rise time, Δt. During the falling phase of current in the coil the rate of change in magnetic flux would be zero minus the maximal value, divided the fall time, Δt. Taking Δt as the time for rising or falling phases, one can apply Faraday’s law to find the average EMF generated around a circular loop of radius r during either rising or falling phases as

(6) $$\frac{-\Phi {\left(z\right)}_{\text{max}}}{\mathrm{\Delta t}}=-\frac{B_{z\max}\left(z,0\right)\pi r^{2}}{\mathrm{\Delta t}}\left(1-\frac{r^{2}}{2r_{0}^{2}\left(z\right)}\right).$$

Finally, the signed magnitude of the induced electric field $\vec{E}$ in units, for example, of V/m is the total EMF divided the total path length, 2πr,

(7a) $$\begin{array}{l} E\left(z,r\right)=-\frac{B_{z\max}\left(z,0\right)\pi r^{2}}{2\mathrm{\pi r}\cdot \mathrm{\Delta t}}\left(1-\frac{r^{2}}{2r_{0}^{2}\left(z\right)}\right)=-\cdot \frac{\mu_{0}}{2}\frac{R^{2}I_{\text{max}}}{{\left(z^{2}+R^{2}\right)}^{3/2}}\cdot \frac{r}{2\mathrm{\Delta t}}\left(1-\frac{r^{2}}{2r_{0}^{2}\left(z\right)}\right)\;\text{or}\;\\ E\left(z,r\right)=-\frac{\mu_{0}}{4\mathrm{\Delta t}}\frac{R^{2}I_{\text{max}}\;r}{{\left(z^{2}+R^{2}\right)}^{3/2}}\left(1-\frac{r^{2}}{2r_{0}^{2}\left(z\right)}\right),\text{for}\;0<r<r_{0}. \end{array}$$

To estimate the voltage gradient beyond r_0_, assume that dΦ(z, r) ≈ 0 for r > r_0_, so that Φ(z, r) = Φ(z, r_0_). Then using Equation (5) it is straightforward to show that

(7b) $$E\left(z,r\right)=E\left(z,r_{0}\right)\frac{r_{0}}{r},\text{for}\;r>r_{0}.$$

This expression can be further simplified by specifying the rate of current change in the coil as $\left(\frac{\mathrm{dI}}{\mathrm{dt}}\right)\approx \frac{I_{\text{max}}}{\mathrm{\Delta t}}$, using normalized radial distance $\hat{r}=\frac{r}{R}$ and axial distance $\hat{z}=\frac{z}{R}$, and the linear approximation for zero crossing radius, ${\hat{r}}_{0}=a\hat{z}+b$, as shown in Figure 4. Then for any sized coil with ${\hat{r}}_{0}=a\hat{z}+b$

(7c) $$E\left(\hat{z},\hat{r}\right)=-\cdot \frac{\mu_{0}}{4}\left(\frac{\mathrm{dI}}{\mathrm{dt}}\right)\frac{\;\hat{r}}{{\left({\hat{z}}^{2}+1\right)}^{3/2}}\left(1-\frac{1}{2}\frac{{\hat{r}}^{2}}{{\hat{r}}_{0}^{2}}\right),\mathrm{for}\;0\leq \hat{r}\leq {\hat{r}}_{0}\;$$

(7d) $$E\left(\hat{z},\hat{r}\right)=E\left(\hat{z},{\hat{r}}_{0}\right)\frac{{\hat{r}}_{0}}{\hat{r}},\;\mathrm{for}\;\hat{r}>{\hat{r}}_{0}.$$

**Figure 5:**
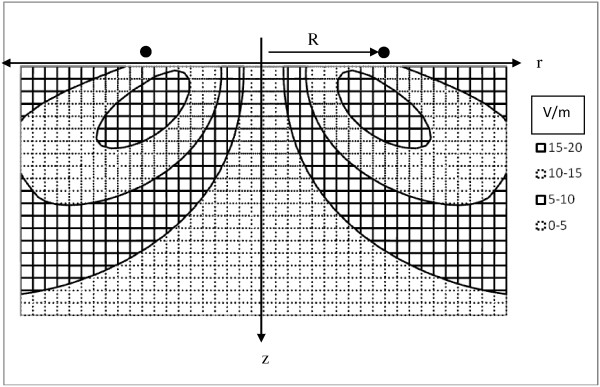
**Contour plot of the electric field induced by a neurostimulating coil (solid dots) in a central plane below a one-turn coil of radius R.** The coil is parallel the surface (z = 0) of a semi-infinite volume of homogenous tissue. Field vectors are perpendicular to the plane of the page. Contour intervals are 5 V/m apart.

Equations (7) give a simple, closed form, analytical expression for the electric field induced by magnetic stimulation at any particular depth, z, beneath the plane of the coil in a semi-infinite tissue volume. The direction of the electric field vector is perpendicular to radius r(z), at any particular z-level. Now it is possible to plot and visualize the strength of electric fields produced by simple circular coils used for neural stimulation, as shown in Figure 5.

For example, one can compute the magnitude of induced EMF caused by a typical magnetic pulse, for which in practical units the maximal current output I_max_ ≈ 10,000 amperes, Δt ≈ 100 microseconds, giving dI/dt ≈ 10^8^ A/sec [4], with the magnetic permeability constant, μ_0_ = 4π × 10^−7^ V · sec /(A · m). Current in the coil is typically produced by repeated capacitor discharges, which are damped by the inductance of the coil and opposed also by the small coil resistance. In such a discharge circuit the current rises quickly to a maximal level and then decays quickly [28] the rise time Δt_1_ is on the order of 100 microseconds and the fall time Δt_2_ is on the order of 200 microseconds [28], leading to reasonable estimates of (dI/dt).

Figure 5 shows the predicted strength of the magnetic field in Volts/meter given by Equations (7c) and (7d). The induced voltage gradients and eddy currents flow parallel to the surface in circles concentric with the coil, and opposite in direction to the direction of current increase in the coil, as expected from Faraday’s Law. In this simple system there are no insulating boundaries that obstruct current flow and no accumulation of surface charge at insulating boundaries [4]. The resistivity of the tissue of the volume conductor in this model is also homogeneous. The results are quite similar to those reported by Roth and Basser [18] and by Tofts [16] using much more elaborate methods.

The direction of E depends on the direction of current flow in the coil and also on whether the current is increasing or decreasing. If the B-field is rising to its maximal value the induced EMF will be negative in the sense of the right hand rule and Equation (6). When the B-field is falling from its maximal value the direction of the induced EMF will be reversed. The results in Figure 5 are for a single coil with a single turn. For multi-turn coils the current derivative term (dI/dt) is multiplied by the number of turns. As explained subsequently, the same approach can be applied to double figure of eight coils by doubling the induced field in the region of overlap.

### Part two: interaction of neurons with induced electric fields

#### Neuron anatomy and physiology

Anatomically typical nerve cells, or neurons, are most unusually shaped, having a cell body at one end that is perhaps 25 micrometers in diameter and a very long, thin arm or projection of membrane covered cytoplasm known as the axon that is roughly one micron in diameter and can span distances of one thousand to one million micrometers in some cases [29]. Excitation of a nerve cell means the initiation of a self-propagating wave of depolarization known as an action potential, usually starting at one end near the cell body and continuing at speeds of several meters per second down the length of the axon. This special anatomy and physiology allows neurons to send signals at high speed over macroscopic distances from one part of the body to another [30].

Normal neurons are polarized in the resting state by a charge difference across the outer membrane of the cell of (inside negative, outside positive) of approximately −85 mV [30]. In order for an action potential to be generated the transmembrane potential at a particular site on the surface of the cell must be brought from the resting level of −85 mV to a threshold level near −55 mV, at which voltage sensitive sodium channels in the membrane open to allow depolarizing current to flow in the form of charged Na^+^ ions. This current eliminates the transmembrane charge difference locally and triggers depolarization of adjacent membrane, sending a propagated signal along the length of the axon [31].

#### Cell bodies are not stimulated directly

**Figure 6:**
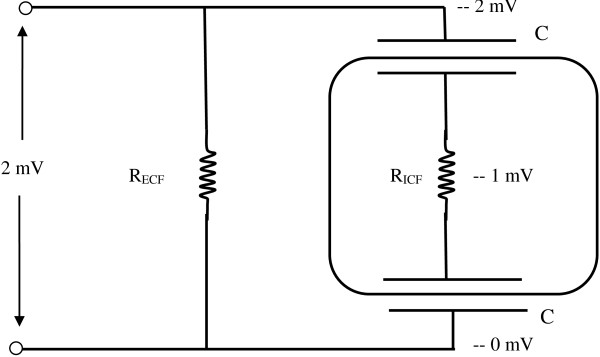
**Equivalent circuit model of a typical neuron cell body, drawn here as a cube, in a potential gradient field.** R_ECF_ is the extracellular fluid resistance. R_ICF_ is the intracellular resistance. Insulating up-field and down-field cell membranes act as series capacitors, C. Half the total voltage difference across the cell appears across each membrane. In magnetic stimulation a voltage gradient on the order of 10 microvolts/micrometer × 20 micrometers or 2 mV appears across the cell. One side of the cell is depolarized and the other side is hyperpolarized by half this amount or 1 mV, which is insufficient to stimulate the cell.

Figure 6 shows a model of a cell body having a diameter on the order of 25 micrometers [29]. The cell body is modeled as a small volume conductor providing electrical resistance surrounded by an insulating membrane, opposite sides of which act a capacitors when an electrical potential difference appears across the cell body. The cell is electrically in parallel with surrounding extracellular fluid (ECF), which provides a parallel path for electric current. According to basic electronic theory [32], the sudden appearance of an electric field will stimulate current flow. Stimulating current density depolarizes membrane capacitances to change the membrane potential. The membrane capacitors become charged, each to one half the transcellular potential difference. The time constant for charging the membrane capacitance, equal to the time required to achieve 63% of a complete charge from a cold start, is τ = R_ICF_C/2, the product of the intracellular fluid resistance and net capacitance, or

(10) $$\tau =\rho \frac{s}{s^{2}}\cdot \frac{C_{m}s^{2}}{2}=\frac{\rho C_{m}s}{2},$$

for in a cubical model of a cell body having sides, s, with cell fluid resistivity, ρ, and specific capacitance, C_m_, per unit area of cell membrane. For ρ = 200 ohm-cm [33] and C_m_ = 1 microfarad per square centimeter [30] and s = 25 × 10^−4^ cm [29] the time constant, τ, is here about 0.25 microseconds. Magnetically induced pulses last about 100 microseconds [28] so there is plenty of time to fully charge the cell body after a magnetically induced E-field appears across it.

The full magnitude of the potential appearing across the cell, however, is not significant physiologically. In the case of Figure 5, taking the maximal gradient as 25 V/m, the total voltage developed across the cell is only 25 μV/μm × 25 μm = 625 μV or 0.6 mV, a far cry from the 30 or so mV needed to depolarize one side of the cell from the resting membrane potential to the threshold potential. This same reasoning also applies to larger cell bodies, like pyramidal cells in the brain. In the same vein it is obvious that electric fields that appear at right angles to axons in nerve fiber tracts cannot induce a threshold membrane potential change, since the diameters of axons are on the order of only 1 micrometer. This conclusion agrees with the prior work of Nowak and coworkers [34,35].

#### Axons spanning several millimeters are the sites of magnetic stimulation

**Figure 7:**
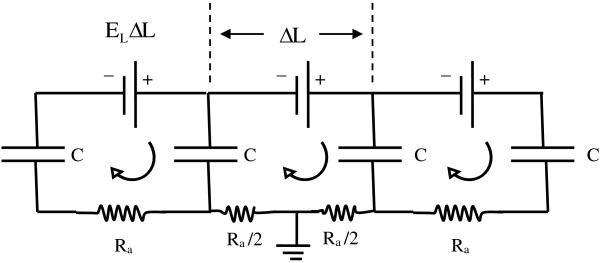
**Simplified model of a non-myelinated axon in a longitudinal voltage gradient E**_**L**_**, directed along the axon.** Voltage sensitive ion channels (not shown) are closed during subthreshold stimulation. If “batteries” E_L_ΔL appear at time zero, then currents will flow in loops shown to charge capacitors with the short time constant R_a_C. For n segments a steady state voltage n ⋅ E_L_ΔL = E_L_ ⋅ L will appear between ends separated by R_tot_ = nR_a_ and with E_L_ ⋅ L/2 across each capacitance, C.

A better hypothesis to explore is that axons are the sites of magnetic simulation. Because of their unusual length-to-width ratio, axons can span distances of 10 mm or more. The potential difference induced by an E-field of just 5 Volts/m or 5 mV/mm over a 10 mm distance equals 50 mV, adequate to stimulate an action potential. Figure 7 illustrates how this situation could occur in terms of a classical cable model [14] describing the electrical properties of an axon. First non-myelinated axons, and then myelinated axons, will be considered.

This model represents successive short segments of the axon as a chain of leaky capacitors having voltage sensitive ion channels, and connected in parallel by extracellular and intracellular resistance elements. Here each short segment of the axon can be modeled as two ring-shaped capacitors linked by the internal resistance, R_a_, of the axoplasm in the segment, as shown in Figure 7. During sub-threshold stimulation (before voltage sensitive ion channels open) this electrical arrangement is very similar to the cell model of Figure 6, with a very short, sub-microsecond time constant for charging. This means that all segments of small length, ΔL, will come up to the full gradient voltage E_L_ ΔL very quickly. When the membrane capacitances along the cable model became fully charged, then the steady-state potential difference between the two widely separated points along the cable model, P_1_ and P_2_ , would be

$$\Delta V_{L}=-\int_{P_{1}}^{P_{2}}E_{L}\mathrm{dL},$$

where *E*_*L*_ is the scalar component of the electric field along the path of the axon, and points P_1_ and P_2_ are points where the axon enters and exits the induced voltage field. In this scenario ± 0.5 ΔV_L_ will appear very quickly across the axonal membrane segments at P_1_ and P_2_.

**Figure 8:**
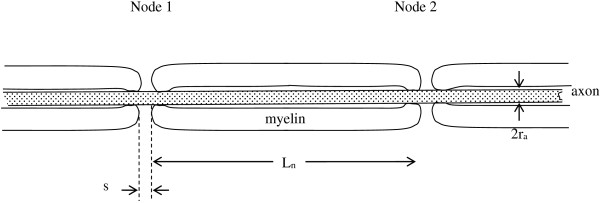
**Schematic longitudinal section of a myelinated axon.** The width of each node of Ranvier is denoted s. The distance between nodes is denoted L_n_. This sketch is foreshortened in the axial dimension. Anatomically L_n_/s ~ 1000.

Many fiber tracts in the central nervous system and in the peripheral nervous system are composed of myelinated axons. Indeed, the majority of the axons in vertebrate nervous systems are wrapped with insulating layers of back-to-back cell membranes called myelin. Myelin forming Schwann cells in the peripheral nervous system or oligodendrocytes in the central nervous system wrap around the axon multiple times to create laminated layers of insulating cell membrane, as shown schematically in Figure 8. Periodic short gaps in the myelin sheath along the axons having width, s, approximately 0.3 to 1 micrometer, are the Nodes of Ranvier, where the density of transmembrane channels carrying inward sodium current is high and where transmembrane action potentials are initiated. Immediately adjacent to the nodes themselves on either side in the axial dimension are the paranodal regions, where myelin is tightly attached to the underlying axonal membrane. Since myelin inhibits the conduction of ionic current, the action potential tends to jump from one node to the next along the longitudinal axis of an axon. This process of jumping, or “saltatory conduction”, boosts the speed of propagation of action potentials along myelinated axons to tens of meters per second, rather than tens of centimeters per second typical of unmyelinated axons [5].

The spacing, L_n_, between nodes of Ranvier in the brain is about 300 micrometers [36], and the axon diameter is about 0.5 micrometers, the gap, s, is about 0.3 micrometers. In turn, the time constant for charging segments of a myelinated axon is, in keeping with Equation (10),

(11) $$\tau =\rho \frac{L_{n}}{\pi r_{a}^{2}}\cdot \frac{C_{m}2\pi r_{a}s}{2}=\frac{\rho L_{n}C_{m}s}{r_{a}},$$

or about 2.4 microseconds. This means that after about 5 microseconds the membrane sections are almost fully charged and the whole voltage gradient appears between the ends of a myelinated axon segment. Since the rise or fall times, Δt, for magnetic pulses are approximately 100 microseconds long, there is plenty of time to charge the membranes of myelinated axons, as well as non-myelinated ones, to the full gradient potential, ΔV_L_, created by magnetic induction.

**Figure 9:**
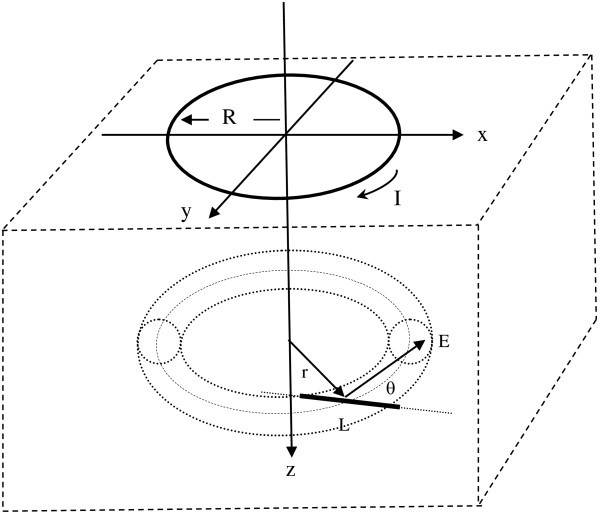
**Axon penetrating a torus of induced voltage and current.** Heavy line represents the axon segment of length, L, which transects a local field of induced EMF at angle θ.

To appreciate the magnitude of ΔV_L_, consider a nerve axon having length, L, that passes through the torus of magnetically induced current and voltage in three dimensions. Let the midpoint of length, L, be located at coordinates z, r (Figure 9), where the induced voltage gradient is $\vec{E}\left(z,r\right)$. The axon makes angle θ in three dimensions with the induced electric field vector $\vec{E},$ which itself is perpendicular to r.

In this context if axon segment, L, is represented as a series of incremental vectors, $d\vec{L}$, then

(12a) $$\Delta V_{L}=-\int_{0}^{L}\vec{E}\cdot d\vec{L}\approx \bar{E}\cos\theta ,$$

where $\bar{E}$ denotes the mean value of the induced electric field along L. Assuming temporarily that a reasonable estimate of $\bar{E}$ for an axon intercepting the center circumference of the torus at z, r (the midpoint of L) is the local point value $\vec{E}\left(z,r\right)$, given by Expressions (7), the absolute value of voltage appearing along the length, L, of axon is

(12b) $$\left|\Delta V_{L}\right|=E\left(z,r\right)\;L\cos\theta$$

with the sign depending on the direction and the temporal phase of the magnetic pulse. Expressions (12) give the axial voltage difference along the length of the axon intercepting the torus of induced voltage and current. The axon membrane between entry and exit points P_1_ and P_2_ will be either hyperpolarized or depolarized by amount.

(13) $$\Delta E_{m}=\pm \frac{1}{2}\left|\Delta V_{L}\right|.$$

**Figure 10:**
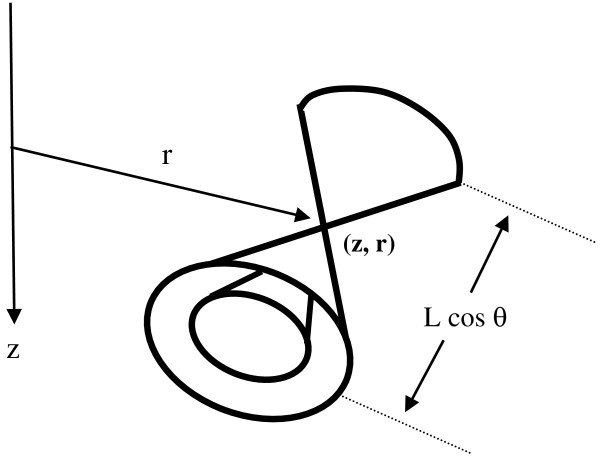
Nested cones of axons with similar induced transmembrane voltages.

Geometrically, for a given projection distance, L cos θ ~ 1 cm, along the axis of induced voltage and current there is a family of possible axon trajectories, forming a nested set of double cones of dimension L cos θ, as shown in Figure 10. The axon segments in each layer would experience the same change in transmembrane voltage in response to a magnetic pulse.

Combining Equations (7), (12), and (13) with r_0_ defined as in Figure 2, gives a simple analytical expression to describe the effect of magnetic stimulation on the transmembrane potentials of affected neurons in terms of the rate of change in coil current, dI/dt, the distance from the plane of the coil in units of coil radius ($\hat{z}=z/R$), the normalized radial distance $\hat{r}=r/R$ from the z-axis through the center of the coil, the length, L, of the axon segment in the induced electric field, and the angle, θ, of the axon segment with respect to the field.

(14a) $$\left|\Delta E_{m}\right|=\frac{\mu_{0}}{8}\left(\frac{\mathrm{dI}}{\mathrm{dt}}\right)\frac{\;\hat{r}}{{\left({\hat{z}}^{2}+1\right)}^{3/2}}\left(1-\frac{1}{2}\frac{{\hat{r}}^{2}}{{\hat{r}}_{0}^{2}}\right)\cdot L\cos\theta ,\mathrm{for}\;0\leq \hat{r}\leq {\hat{r}}_{0},$$

(14b) $$\left|\Delta E_{m}\right|=\frac{\mu_{0}}{8}\left(\frac{\mathrm{dI}}{\mathrm{dt}}\right)\frac{\;{\hat{r}}_{0}}{{\left({\hat{z}}^{2}+1\right)}^{3/2}}\;\frac{1}{2}\;\frac{{\hat{r}}_{0}}{\hat{r}}L\cos\theta ,\mathrm{for}\;\hat{r}>{\hat{r}}_{0}.$$

**Figure 11:**
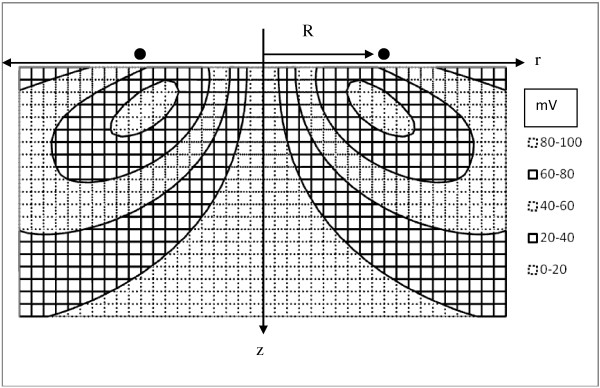
**Contour map of predicted change in axon transmembrane potential in response to magnetic stimulation by a single circular coil of radius, R, at the body surface (z = 0).** The horizontal axis represents radial distance from the z-axis. The vertical axis represents depth into a semi-infinite volume of tissue. The origin is located at the center of the coil. The width of the map is two coil diameters. The height of the map is one coil diameter. The contour interval is 20 mV. In three dimensions zones of equivalent neural stimulation form nested toroidal rings, having a major radius roughly equal to coil radius and increasing slightly with depth. The zone of effective neural stimulation is roughly one half coil diameter in depth for this standardized test case (dI/dt = 10^8^ A/sec, one turn coil, Lcosθ = 1 cm).

Figure 11 shows a contour plot of the absolute value of the change in membrane potential vs. tissue depth and radial distance from the z-axis for magnetic stimulation of axons for a standard model. Contour intervals are 20 millivolts. The rate of change in current in a 1-turn coil is 10^8^ A/sec. Distances are normalized to the radius, R, of the coil. The projection distance, L cos θ, is 1 cm. The values of the contours can therefore be interpreted as millivolts of membrane potential change per centimeter of projection distance along the axis of the induced field. The plotted values are also proportional to the number of turns of the coil and to the peak coil current. Axons travelling through the indicated regions of space more or less parallel with the induced electric field (into and out of the page) will be stimulated to the extent shown in Figure 11. In three dimensions the stimulation zones for a simple circular coil are toroidal or donut shaped.

**Figure 12:**
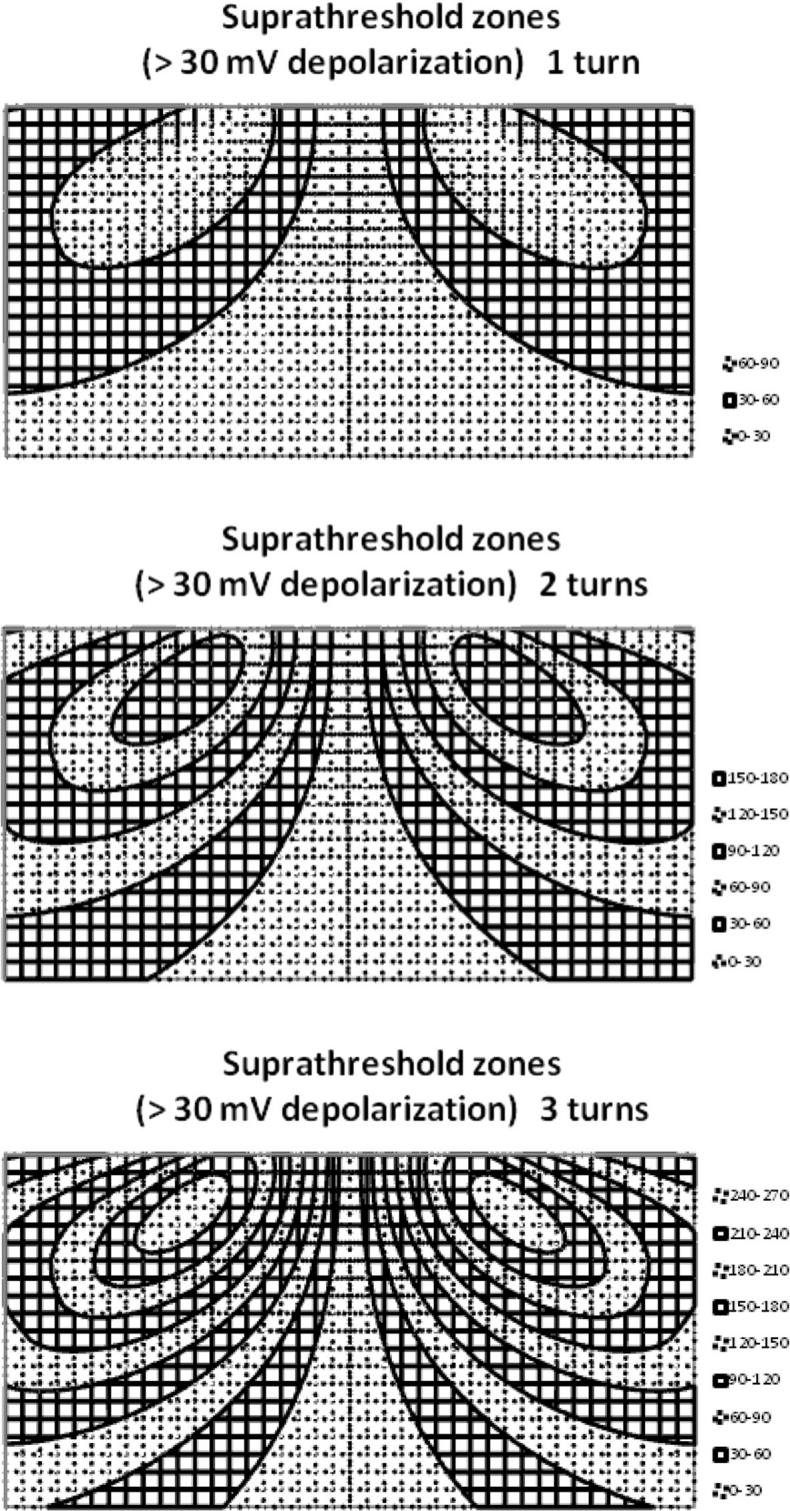
**Contour maps showing modulation of the volume of the target toroid for a simple, circular coil with increasing strength, n, of stimulation, compared to the standard model (n = 1).** The multiplier, n, applies to either the number of turns in the coil, or alternatively the fold increase in dI/dt, or the fold increase in the projection distance, L cos θ, when other variables are held constant. The width of each contour map is two coil diameters, and the height of each map is 1 coil diameter. Legends show maximal transmembrane voltage changes in millivolts.

The ranges over which neurostimulation would occur depend upon the difference between resting membrane potential and threshold membrane potential of the axon. For a typical case [30] resting membrane potential is about −85 mV, inside negative with respect to outside, as would be measured with intracellular microelectrodes, and threshold membrane potential is about −55 mV. Hence a depolarization to the extent of 30 mV would be required to trigger an action potential. This level lies between the 20 and 40 mV contours in Figure 11. The present analysis suggests that the stimulation zones for the standard model extend about one half coil diameter deep into the tissue, depending on coil current intensity. This volume of tissue can be imagined as a donut or torus having a major diameter roughly that of the coil itself. If the number of turns in the coil is increased within limits that do not add too much current limiting resistance, if the projection span of the axon along the E field axis is increased, or if the slew rate of the peak current is increased; then the volume of the target toroid increases as shown in the contour maps of Figure 12. The maximal transmembrane voltage change within the target volume increases linearly, as expected. The depth of the target volume increases much more gradually, however, roughly with the square root of the boosted parameter. Thus achieving stimulation at depth remains a challenge, especially for focused stimulation.

#### Figure of eight coils

**Figure 13:**
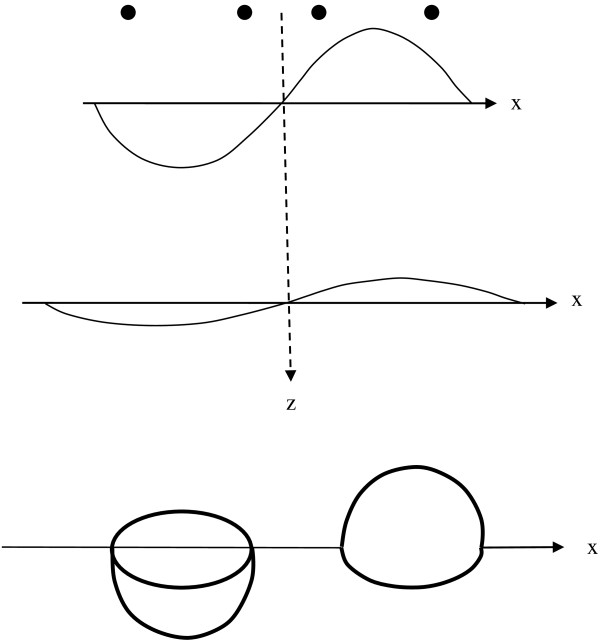
**Joint B-fields of figure of 8 coils (top) and bowl metaphor (bottom).** Figure of eight coils with counter-rotating currents are aligned with the x-axis. B-fields from the two coils and their time derivatives are opposite in sign. As they rise and fall in time counter-rotating eddy currents are induced in underlying tissue which reinforce at points near the z-axis.

Figure of eight coils have been used empirically for many years to boost the effectiveness of magnetic stimulation [17,22,37] and to concentrate the volume of stimulation in a more easily defined region of space. A loop of low resistance wire or metal ribbon is folded into a figure of eight to create counter rotating current loops, side-by-side near the body surface. The individual z-axis magnetic fields from the two coils, B_z_, are opposite in sign and may interact as sketched in Figure 13. The forgoing analysis of individual circular coils can be applied with modification to the figure of eight coils, here in a semi-quantitative way. B_z_-fields add algebraically in the vicinity of the z-axis, but remain largely undisturbed elsewhere by the presence of the other coil. One can imagine the domes of instantaneous magnetic field strength as two bowls placed side-by-side, one inverted, with the complete rims of both bowls in the same x-y plane (Figure 13, bottom).

Helpful reinforcing effects are possible in simple cases without severely restricting lateral boundaries. In a semi-infinite space a double torus of induced voltage and current is created. As the absolute intensities of the paired B_z_-fields rise and fall in time there are counter rotating induced currents that can combine and reinforce the voltage gradient near the common z-axis. As coil current rises and falls the heights of the bowls rise and fall. If current reverses direction, the bowls flip vertically. Parallel components of induced current near the z-axis add to reinforce each other. At a depth of one third to one half coil radius (see below) a sweet spot exists where induced voltage gradients are nearly double what they would have been at the same z-level for a single coil [37]. In the far field the overlapping B-fields near the z-axis cancel, but only in the zone of overlap. Elsewhere around the circumferences of the individual loops of the figure of eight, circular symmetry prevails and eddy currents are still induced, as they would be for a single coil.

**Figure 14:**
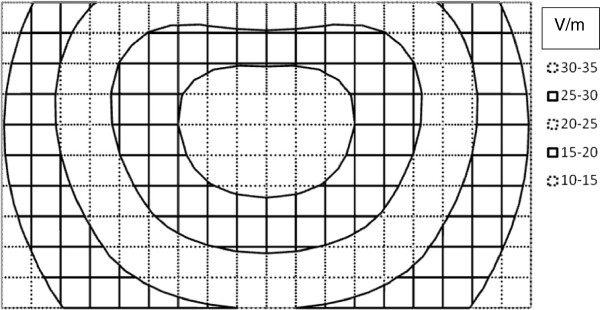
**Contour map of joint induced voltage gradients for figure of eight coils in a region of interest close to the z-axis perpendicular to the body surface and passing through the midpoint between coil centers (crux of the figure of eight).** In this special case results for two simple circular coils (details as in Figure 5) are superimposed to give the joint field in the region of interest ± 0.9 coil radius from the z-axis to estimate the net induced EMF. The width of the contour map is 1.8 coil radii and the height is one coil radius. Contour intervals are 20 V/m apart. The target region of stimulation is centered about 0.4 coil radii from the surface plane.

Quantitatively one can estimate the magnitude of the joint induced current field near the vertical plane through the centers of the two coils by superposition of the induced currents for a single coil in the region of interest including ± 0.9 coil radii, R, from the z-axis. The result is shown in Figure 14. Here the two coils are separated in the x-dimension by 10 percent of the individual coil diameter. A target shaped region of high current density is created with peak intensity approximately 40% of the coil radius from the surface plane (z = 0). This result represents a partial solution to the targeting problem using figure of eight coils.

**Figure 15:**
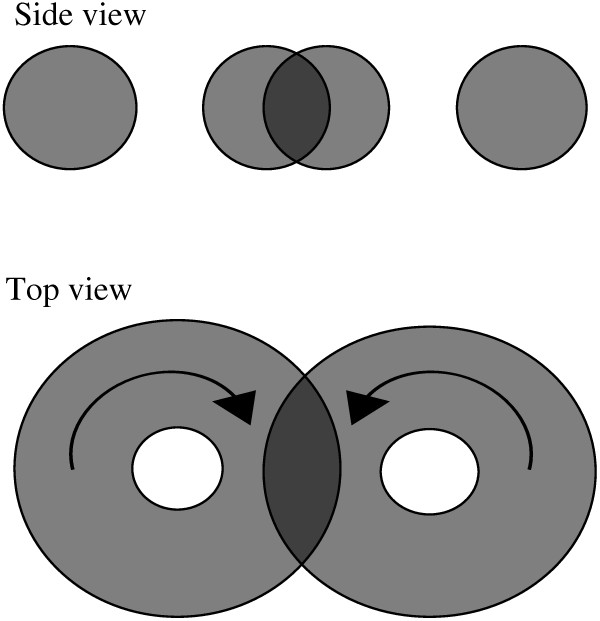
**Joint induced voltage and current fields of figure of eight coils in a semi-infinite uniform volume conductor.** A spindle shaped volume of overlapping induced EMF is created.

The figure of eight concept can be used to create a more focused zone of stimulation, as indicated conceptually by the darker shading in Figure 15 and the contour plot of Figure 14, provided that the current, I_max_, is adjusted so that the single coil stimulus never exceeds threshold, but twice that value does. The focal region will be roughly spindle shaped and correspond to the overlapping region for two toroidal volumes of high induced EMF from either coil. This scheme provides a practical solution to the problem of focusing magnetic neurostimulation and agrees with the prior results of Ravazzini [26]. This concept can be extended to 4 coil fields but vector addition and cancellation limit the boost in net induced EMF from two to four coils to a factor of no more than the  . The reinforcing double toroidal patterns of induced current flow predicted by the present compact analysis are quite similar to those found by Wagner et al. [25] using much more elaborate computational methods. The maps of induced current predicted here are also similar to those described by Pascual-Leone [5]. Figure of eight coils produce neurostimulation that is roughly twice as strong as that produced by single coils and confined to a much smaller, targeted volume of tissue.

## Discussion

Axons running in many directions, but not directly parallel to the z-axis of an electromagnetic coil carrying rapidly changing current can be depolarized or hyperpolarized by induced electric fields at depths up to one coil diameter from the plane of the coil. Both myelinated and non-myelinated axons can be stimulated. The sites of greatest membrane depolarization are located toward the ends of the axon segments traversing the region of induced voltage and current. The target volume for magnetic stimulation takes the shape of a toroid centered along the z-axis with major radius similar to that of the current carrying coil and its minor radius varying in size, depending on the coil current. When figure of eight coils are used there is the opportunity to create spindle shaped target volumes of stimulation, centered on the z-axis at a depth of about one third to one half coil radius.

In the forgoing analytical treatment many simplifying assumptions have been introduced to make the mathematics more tractable and useful, realizing than in any particular biological experiment there is some lack of precision in specifying geometric distances and physiological conditions. Hence, only the most dominant terms in the mathematics need to be considered in a biologically satisfactory solution to the targeting problem. For example, the position of a coil in a clinical stimulation protocol can only be specified within about one millimeter. The thickness of a practical coil is on the order of several millimeters, perhaps better characterized as a bundle of one dimensional wire loops, blurring the idealized fields calculated for any single loop. The positions of underlying nerves and brain structures vary from subject to subject and typically cannot be known exactly in a particular treatment situation or experiment. Similarly, the intracellular and extracellular sodium and potassium ion concentrations that determine resting and threshold membrane potentials can vary among subjects, the intracellular values being especially hard to measure. Additionally there are motion artifacts, including subtle movement in time and space with breathing, fidgeting, as well as any muscle movement caused by nerve stimulation itself.

Accordingly, the positions of biologic structures with respect to magnetic and electric fields can only be specified within a millimeter or two at best. For such reasons over-precise prediction is a fool’s errand. What is more useful is the ability to predict within, say, 10 percent, the locations and directions of axons likely to be stimulated by a given practical apparatus, and especially the ability to visualize in three dimensions the locations and orientations of nerve axons likely to be stimulated by a given coil and current intensity. Equations (14a) and (14b) provide a compact, closed form solution for the effects of magnetic neurostimulation in terms of the dominant variables: the slew rate of coil current, the axial and radial distances of the target axon from the center of the coil, the length of the axon within the induced electric field, and the angle of the axon in space. This kind of predictive capability has not been available heretofore.

The theory presented here is validated by experimental observations. When using a single circular coil to stimulate the median nerve in the wrist, Maccabee et al. [37] found the most sensitive position for stimulation the nerve was located beneath the middle circumference of the coil in the position of maximal toroidal current. However when figure of eight coils were used, the most sensitive position for stimulation of the nerve was located at the midpoint between the two loops of the coil. Rudiak and Marg [38] used a clever approach to estimate the effective depth of magnetic brain stimulation in human subjects. For figure of eight coils with 10 cm diameter (5 cm radius) loops, the focal depth of stimulation was between 1.8 and 2.1 cm, or between 36% and 42% of coil radius, very close to that predicted by the present analysis (Figure 14).

## Conclusion

Magnetic neural stimulation is a remarkable and subtle phenomenon, able to penetrate highly resistive skin and bone with ease and stimulate underlying nerve fibers. This paper presents a condensed synthesis of electromagnetic theory and cable theories of axon physiology to better inform further development and clinical practice of magnetic neurostimulation, including transcranial magnetic stimulation. With knowledge of the critical variables and a little imagination it is possible to visualize in three dimensions the best way to arrange and orient surface coils to achieve stimulation within a defined volume of underlying tissue.

## Competing interests

The author declares that he has no competing interests.
